# Tobacco tax and price in the developed and developing countries in the World

**DOI:** 10.1016/j.dib.2018.06.100

**Published:** 2018-07-03

**Authors:** Mina Riahi, Hosein Rohani, Naser Rajabi, Mohammad Bidkhori

**Affiliations:** a Department of Health, Shahrekord University Of Medical Science, Shahrekord, Iran; b Student research committee, Esfarayen Faculty of Medical Sciences, Esfarayen, Iran; c Department of Epidemiology and Biostatistics, Isfahan University of Medical Sciences, Isfahan, Iran; d Department of Public Health, Neyshabur University of Medical Sciences, Neyshabur, Iran

**Keywords:** Tobacco, Tobacco affordability, Tobacco tax, Human Development Index

## Abstract

An ecologic study was conducted on 177 countries which the information of tobacco tax and price and also Human Development Index (HDI) was available in 2014. In this study, the relationship between HDI and four reported indexes by World Health Organization (WHO) was studied. These four indexes included: Tobacco affordability, Taxes as a percent of price of the most sold brand (total tax), Price of a 20 cigarette pack of the most sold brand international dollars at purchasing power parity (Price_ppp) and Price of a 20 cigarette pack of the most sold brand in US$ at official exchange rates (Price_US$). The data of HDI and tobacco were mined from WHO and United Nations Development Programme sites respectively. To study the correlation between HDI and the variables of this study, Pearson correlation coefficient was used and also Linear Regression Analysis was used to study the relationship between HDI and the variables of the study. According to the findings of the linear regression analysis, there was a significant relationship between HDI and total tax (*B* = 0.81, CI 95%: 0.63–0.99) and tobacco affordability (*B* = --0.35, CI 95%: --0.42 _ --0.28). There was also a significant relationship between HDI whit price-ppp (*B* = 9.44, CI 95%: 7.13–11.75) and price-US$ ;(*B* = 11.97, CI 95%: 9.71–14.23). According to the findings of this study, less developed countries devote less tax on tobacco. Due to the rising trend of the prevalence and also development of non-communicable diseases such as lung cancer in developing countries, policy makers of these countries are required to design stricter policies toward tobacco production and supply as well.

**Specifications Table**

**Table:** 

Subject area	environmental science
More specific subject area	Economics
Type of data	Table and figure
How data was acquired	Secondary data
Data format	Raw and analyzed
Experimental factors	In order to determine the association and correlation between the variables, linear regression and Pearson׳s correlation analyses were performed respectively by STATA 14.
Experimental features	Investigation relationship between human development index (HDI) whit Tobacco affordability, taxes in the price of a cigarette pack (total tax) and Price of a 20 cigarette pack of the most sold brand
Data source location	Data Obtained from: WHO, United Nations Development Programme
Data accessibility	Data are available from:
	United Nations Development Programme. Human Development Report 2016 [cited 2017 December 13, 2017]. Available from: http://hdr.undp.org/sites/default/files/2016_human_development_report.pdf.
	World Health Organization. WHO report on the global tobacco epidemic 2017 [cited 2017 December 8, 2017]. Available from: http://www.who.int/tobacco/global_report/2013/full_dataset/en/

**Value of the data**

• Recognition of barriers policy makers are faced with to control tobacco consumption is of great significance.
• The findings of the study reveal that environmental, social and economic factors are among the most influential factors on governments׳ actions to control tobacco consumption.
• According to the findings of the study, less developed countries devote less tax on tobacco. Due to the rising trend of the prevalence of non-communicable diseases such as lung cancer in developing countries, the policy makers of these countries are required to design stricter policies toward tobacco׳s production and consumption.
• This study was an investigation about the laws of different countries toward framework convention on tobacco control based on Human Development Index.

## 1 Data

After excluding those countries whose information was unavailable or incomplete, 177 countries were investigated in the present study (Table 1).

**Table 1:** Human Development Index, Tobacco affordability, Taxes as a percent of price of the most sold brand, Price of a 20-cigarette pack of the most sold brand international dollars at purchasing power parity and Price of a 20 cigarette pack of the most sold brand in US$ at official exchange rates in 2014.

**Country**	**Total tax estimate**	**Affordability**	**Price _ppp**	**Price_US$**	**HDI**
Afghanistan	0.03	0.05	1.04	0.35	0.479
Libya	0.08	0.04	5.21	2.38	0.719
Benin	0.09	0.11	2.28	1.02	0.481
Iran	0.11	0.01	1.85	0.57	0.774
Sierra Leone	0.13	0.1	1.95	0.78	0.431
Togo	0.13	0.13	1.79	0.82	0.484
Antigua and Barbuda	0.15	0.02	4.94	2.96	0.784
Paraguay	0.16	0.01	0.64	0.35	0.692
Azerbaijan	0.17	0.02	3.94	1.79	0.758
Lao People׳s Democratic Republic	0.17	0.06	2.9	0.99	0.582
Saint Vincent and the Grenadines	0.17	0.03	3.01	1.85	0.72
Cameroon	0.18	0.07	2.15	1.02	0.514
Guinea-Bissau	0.19	0.09	1.39	0.61	0.421
Ethiopia	0.19	0.12	2.06	0.76	0.441
Iraq	0.19	0.01	1.04	0.43	0.649
Saudi Arabia	0.2	0.01	5.71	2.67	0.845
Qatar	0.2	0	4.01	2.75	0.855
United Arab Emirates	0.2	0.01	4.15	2.72	0.836
Bahrain	0.2	0.01	4.93	2.66	0.823
Saint Kitts and Nevis	0.2	0.02	4.4	2.96	0.762
Gabon	0.21	0.02	3.67	2.04	0.694
Nigeria	0.21	0.04	2.57	1.42	0.525
Malawi	0.21	0.55	6.1	2.01	0.473
Cambodia	0.22	0.04	1.34	0.44	0.558
Oman	0.22	0.01	4.59	2.34	0.795
Liberia	0.23	0.16		0.69	0.427
Dominica	0.23	0.02	2.37	1.57	0.724
Rwanda	0.23	0.13	2.28	0.95	0.493
Cabo Verde	0.24	0.06	3.83	2.18	0.646
Kuwait	0.24	0.01	4.6	2.65	0.799
Angola	0.24	0.04	2.85	2.06	0.531
Mauritania	0.25	0.11	4.68	1.74	0.513
Sao Tome and Principe	0.25	0.06	2.01	1.09	0.565
Guyana	0.25	0.04	2.62	1.45	0.638
Côte d׳Ivoire	0.26	0.09	3.02	1.43	0.466
Tajikistan	0.26	0.09	2.46	1.01	0.625
Turkmenistan	0.26	0.05	7.29	4.09	0.688
Ghana	0.28	0.06	2.42	0.82	0.575
Solomon Islands	0.28	0.2	3.88	4.13	0.514
Niger	0.28	0.21	2.23	1.02	0.351
Mali	0.28	0.16	3.28	1.43	0.438
Nepal	0.28	0.19	4.54	1.37	0.555
Djibouti	0.29	0.07	2.04	1.13	0.47
Trinidad and Tobago	0.3	0.02	5.88	3.6	0.779
Mozambique	0.31	0.15	1.75	0.98	0.414
Chad	0.31	0.11	3.02	1.43	0.394
United Republic of Tanzania	0.31	0.21	5.63	2.12	0.519
Burkina Faso	0.32	0.14	2.39	1.02	0.399
Viet Nam	0.32	0.04	2.44	0.88	0.678
Nicaragua	0.32	0.08	3.8	1.5	0.642
Uzbekistan	0.33	0.05	2.6	0.94	0.697
Central African Republic	0.33	0.28	1.69	1.02	0.347
Armenia	0.33	0.04	3.03	1.48	0.741
Zambia	0.34	0.08	3.23	1.47	0.576
Timor-Leste	0.34	0.11	1.7	1.25	0.603
Senegal	0.35	0.1	2.25	1.02	0.491
Zimbabwe	0.36	0.13	2.51	1.3	0.507
Papua New Guinea	0.36	0.22	7.33	6.54	0.515
Belize	0.37	0.05	4.34	2.5	0.706
Honduras	0.37	0.07	3.45	1.72	0.623
Peru	0.38	0.03	4.02	2.22	0.737
Kyrgyzstan	0.39	0.05	1.69	0.68	0.662
Kazakhstan	0.39	0.01	2.31	1.15	0.793
Bolivia	0.39	0.05	3.09	1.45	0.671
India	0.41	0.14	8.13	2.29	0.615
Congo	0.41	0.04	2.55	1.22	0.59
Namibia	0.42	0.06	6.9	3.74	0.637
Barbados	0.42	0.04	7.27	6.93	0.794
Burundi	0.43	0.32	2.84	1.03	0.406
Leba0n	0.43	0.02	3.52	2.16	0.763
Jamaica	0.43	0.15	12.55	7.1	0.729
United States of America	0.43	0.01	6.23	6.23	0.918
Bahamas	0.43	0.03	7.29	7	0.79
China	0.44	0.02	2.81	1.62	0.734
Mongolia	0.44	0.04	4.25	1.44	0.733
Fiji	0.44	0.08	6.98	4.21	0.734
Swaziland	0.45	0.08	7.73	3.27	0.541
Uganda	0.45	0.1	2.11	0.76	0.488
Gambia	0.46	0.17	2.7	0.71	0.45
Lesotho	0.46	0.24	8.11	3.27	0.495
Democratic Republic of the Congo	0.48	0.18	1.31	0.81	0.425
Russian Federation	0.48	0.01	3.29	1.88	0.805
Grenada	0.48	0.03	4.11	2.78	0.751
Georgia	0.49	0.03	2.59	1.26	0.768
Kenya	0.49	0.08	2.47	1.14	0.55
Guatemala	0.49	0.06	4.22	2.05	0.637
Colombia	0.49	0.02	2.09	1.32	0.724
South Africa	0.49	0.05	5.91	2.97	0.665
Kiribati	0.49	0.26	4.5	4.48	0.586
Myanmar	0.5	0.05	2.61	0.67	0.552
Equatorial Guinea	0.51	0	1.75	1.02	0.582
Comoros	0.51	0.15	2.35	1.36	0.498
Republic of Moldova	0.51	0.05	2.39	1.08	0.701
Algeria	0.51	0.02	2.73	1.08	0.743
Belarus	0.52	0.01	1.57	0.68	0.798
Vanuatu	0.52	0.24	6.23	7.56	0.598
El Salvador	0.53	0.05	4.07	2	0.678
Indonesia	0.53	0.04	4.67	1.58	0.686
Yemen	0.54	0.08	3.14	1.3	0.499
Botswana	0.55	0.04	6.65	3.08	0.698
Eritrea	0.55	0.59	7.94	3.9	0.418
Samoa	0.55	0.1	4.96	4.13	0.702
Malaysia	0.55	0.03	8.36	3.76	0.787
Iceland	0.56	0.02	8.79	10.59	0.919
Suriname	0.56	0.03	4.71	2.73	0.723
Australia	0.57	0.03	11.73	15.9	0.937
Panama	0.57	0.03	7.06	4.25	0.785
Micronesia (Federated States of)	0.58	0.07	2.02	2.12	0.637
Dominican Republic	0.59	0.05	7.47	3.43	0.718
Pakistan	0.61	0.03	1.65	0.48	0.548
Switzerland	0.61	0.01	6.2	9.24	0.938
Tonga	0.61	0.11	5.28	4.68	0.718
Republic of Korea	0.62	0.01	3.01	2.43	0.899
Saint Lucia	0.63	0.03	3.79	2.69	0.735
Albania	0.64	0.04	4.51	1.93	0.762
Japan	0.64	0.01	4.21	4.18	0.902
Brazil	0.65	0.02	3.33	2.54	0.754
Singapore	0.66	0.02	15.35	10.44	0.924
Maldives	0.66	0.03	4.07	2.47	0.701
Palau	0.67	0.04	5.15	5.25	0.783
Uruguay	0.67	0.02	4.19	3.35	0.794
Andorra	0.68			4.68	0.857
Mexico	0.68	0.03	5.62	3.45	0.758
Sweden	0.69	0.01	6.78	8.55	0.909
Cuba	0.7			7	0.773
Morocco	0.7	0.07	5.48	2.34	0.645
Argentina	0.7	0.01	2.69	1.77	0.826
Sri Lanka	0.7	0.12	12.94	4.61	0.764
Canada	0.7	0.02	7.49	8.49	0.919
Luxembourg	0.7	0.01	5.41	6.69	0.896
Ecuador	0.7	0.05	5.59	3.1	0.739
Costa Rica	0.7	0.03	4.29	2.97	0.775
Venezuela	0.71	0.09	16.1	14.32	0.769
Sudan	0.72	0.13	5.47	2.46	0.488
Egypt	0.73	0.03	3.8	1.12	0.688
Netherlands	0.73	0.02	7.77	8.45	0.923
Thailand	0.73	0.03	5.29	2.03	0.738
Mauritius	0.73	0.04	7.62	4.1	0.779
Germany	0.73	0.02	7.04	7.32	0.924
Philippines	0.74	0.02	1.47	0.62	0.679
Austria	0.74	0.01	5.91	6.56	0.892
Portugal	0.75	0.03	7.34	6.02	0.841
Malta	0.75	0.02	8.56	6.42	0.853
Croatia	0.75	0.03	6.22	4.04	0.823
Romania	0.75	0.04	8.59	4.39	0.798
Denmark	0.75	0.01	5.74	7.89	0.923
Tunisia	0.75	0.03	3.94	1.48	0.723
Belgium	0.76	0.02	6.98	7.75	0.895
Lithuania	0.76	0.02	5.34	3.65	0.846
Italy	0.76	0.02	6.62	6.69	0.881
Bangladesh	0.76	0.08	2.63	0.9	0.575
New Zealand	0.77	0.03	11.51	14.43	0.913
Latvia	0.77	0.03	6	4.01	0.828
Czech Republic	0.77	0.02	5.34	3.49	0.875
Estonia	0.77	0.02	6.46	4.68	0.863
Hungary	0.77	0.03	7.73	4.29	0.834
Cyprus	0.77	0.02	6.3	5.35	0.854
Serbia	0.78	0.03	4.17	1.95	0.775
Spain	0.78	0.02	7.15	6.42	0.882
Ireland	0.78	0.02	11.88	12.84	0.92
Montenegro	0.78	0.02	3.59	1.74	0.804
Madagascar	0.8	0.27	3.97	1.22	0.511
Slovenia	0.8	0.02	5.7	4.62	0.888
France	0.8	0.02	8.52	9.37	0.894
Seychelles	0.8	0.04	10.01	6.09	0.781
Poland	0.8	0.03	7.66	4.41	0.852
Greece	0.8	0.02	6.4	5.35	0.865
Ukraine	0.81	0.02	2.12	0.74	0.748
Bosnia and Herzegovina	0.82	0.05	5.29	2.53	0.747
Turkey	0.82	0.03	6.95	3.82	0.764
Finland	0.82	0.01	5.95	7.36	0.893
United Kingdom of Great Britain and 0rthern Ireland	0.82	0.03	10.78	12.69	0.908
Slovakia	0.82	0.02	5.8	3.8	0.842
Chile	0.83	0.02	4.73	2.98	0.845
Bulgaria	0.83	0.04	7.4	3.21	0.792
Jordan	0.83	0.03	3.77	1.69	0.741
Israel	0.84	0.02	7.48	8.75	0.898

**Fig. 1:**
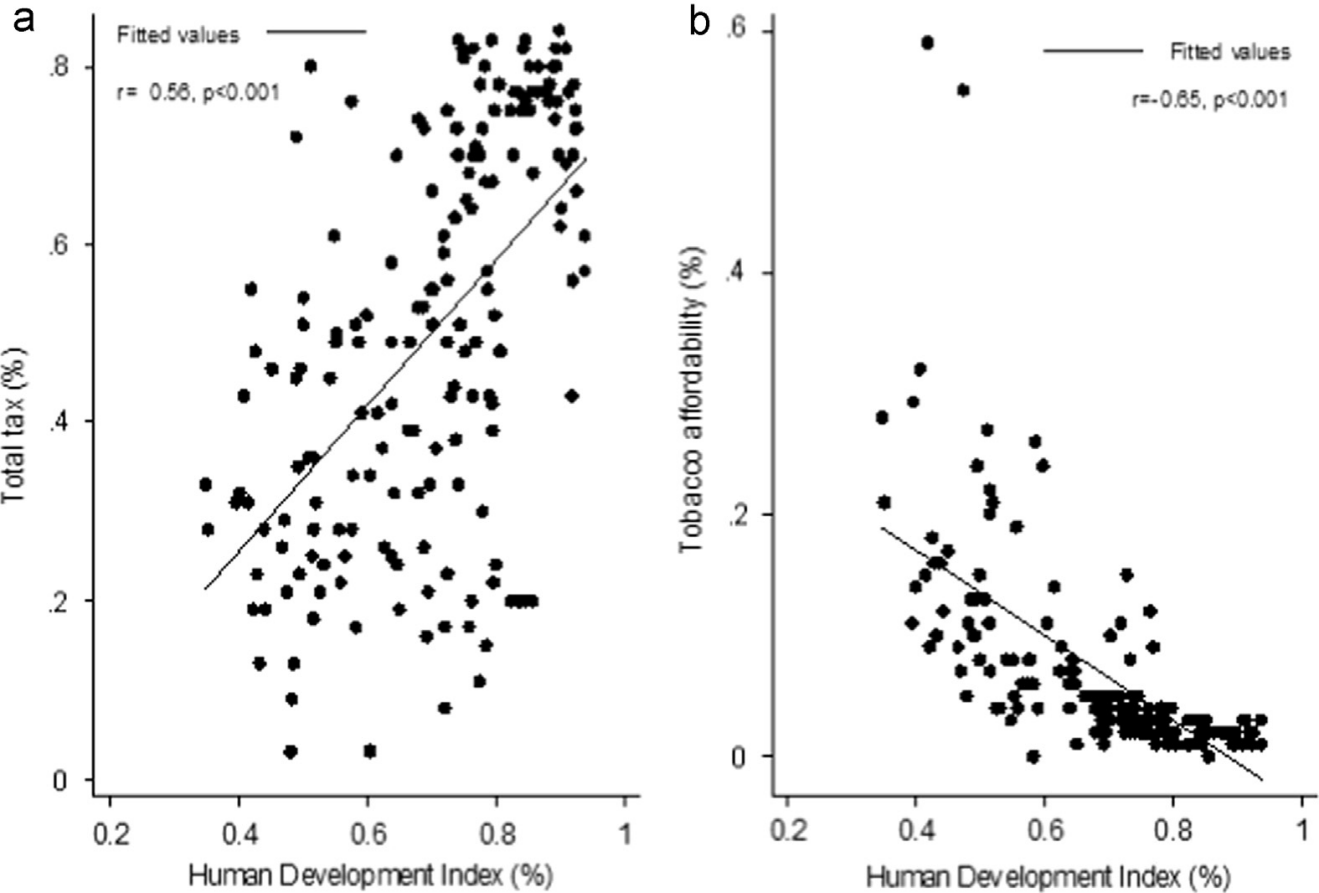
Correlation between HDI and a: taxes in the price of a cigarette pack (total tax), b: Tobacco affordability in 2014.

There was a positive correlation between HDI and total tax which was significant statistically (*r* = 0.56, *p* < 0.001). There was also a negative and significant correlation between HDI and affordability (*r* = −0.65, *p* < 0.001) (Fig. 1).

To study the association between HDI and total tax, linear regression analysis was used. According to the findings of this study, there was a statistically significant relationship between HDI and total tax (*B* = 0.81, CI 95%: 0.63–0.99). In fact, in average when HDI increases for one unit, tax rises proportionally.

According to the linear regression analysis, there was a statistically significant relationship between HDI and affordability (*B* = 0.35, CI 95%: −0.41, −0.28). According to the findings of this study, in average when HDI increases for one unit, affordability decreases. Therefore, countries with higher HDI level possess lower level of affordability (Table 2).

**Table 2:** Effect of HDI on total tax and affordability in 2014.

Independent variable	Dependent variable	B	*p*-Value	95% Confidence Interval
HDI				
	Total tax	0.81	< 0.001	(0.63–0.99)
	Affordability	−0.35	< 0.001	(--042 _--0.28)

**Fig. 2:**
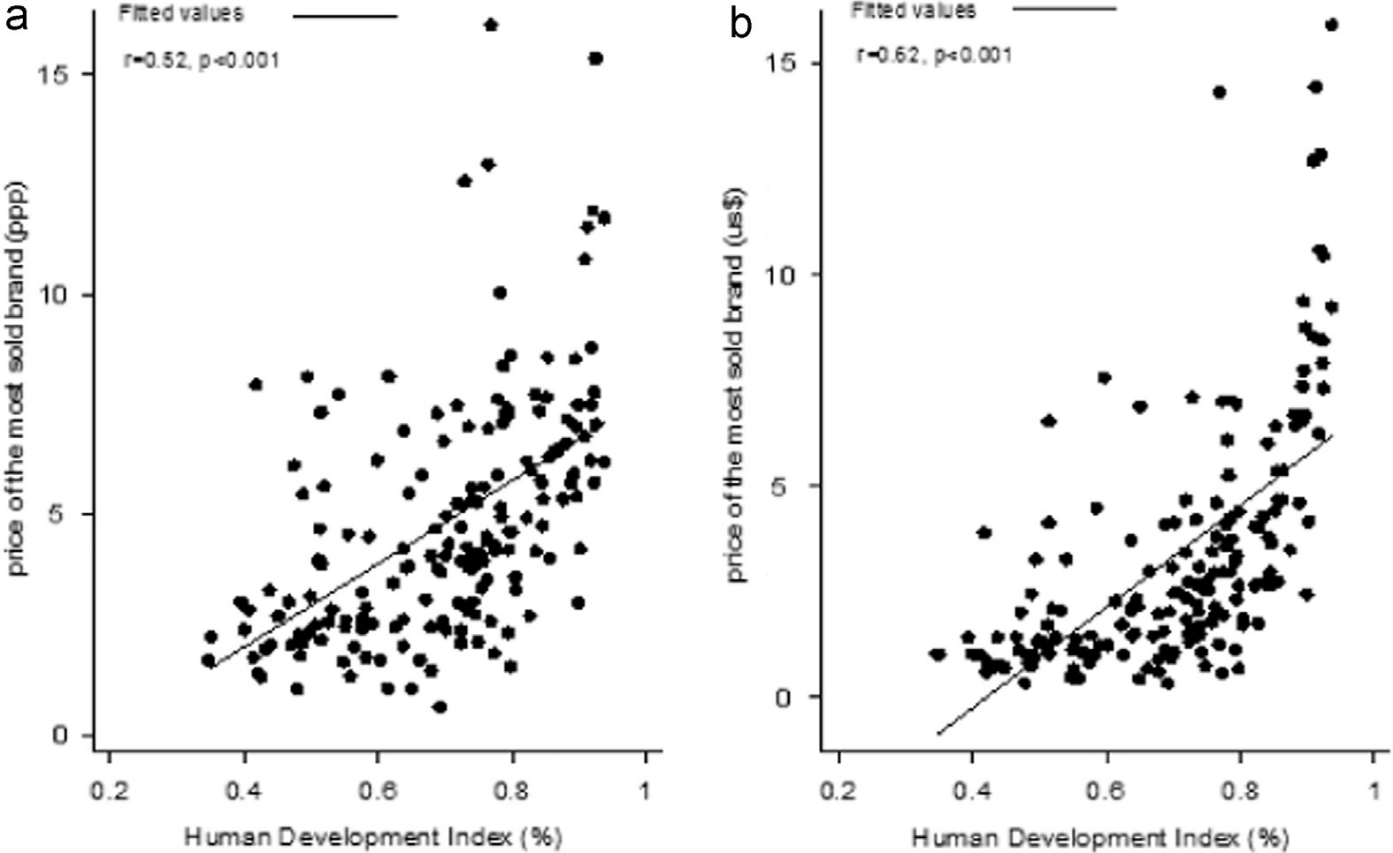
Correlation between HDI and a: price of the most sold brand (ppp), b: price of the most sold brand (US$) in 2014.

In the present study, there was a significant and negative correlation between HDI and price-ppp (*r* = 0.52, *p* < 0.001) and price-US$ (*r* = 0.62, *p* < 0.001) (Fig. 2). As seen in Fig. 2, in countries with lower HDI, less money is required to purchase tobacco products which makes the process of tobacco availability easier. In contrast, in countries with higher HDI, more money is required to purchase tobacco products which impacts the process of people׳s intention and availability tobacco.

Linear regression analysis found a statistically significant relationship between HDI and price-ppp (*B* = 9.44, CI 95%: 7.13–11.75) and price-US$ (*B* = 11.97, CI 95%: 9.71–14.23) (Table 3).

**Table 3:** Effect of HDI on Price _ppp and Price _US$ in 2014.

**Independent variable**	**Dependent variable**	**B**	**p-value**	**95% Confidence Interval**
HDI				
	Price _ppp	9.44	< 0.001	(7.13–11.75)
	Price _US$	11.97	< 0.001	(9.71–14.23)

## 2 Experimental design, materials and methods

### 2.1 Study countries description

Based on the studied conducted, tobacco tax and price are among the influential factors on the fall of tobacco consumption [1], [2], [3], [4], [5], [6]. An ecologic study was conducted. After crossing out those countries whose information was unavailable or incomplete, 177 countries were investigated. In this study, the relationship between HDI and four variables of the study was analyzed. In this study, the relationship between HDI and four reported indexes by World Health Organization (WHO) was studied. These four indexes included: Tobacco affordability, Taxes as a percent of price of the most sold brand (total tax), Price of a 20 cigarette pack of the most sold brand international dollars at purchasing power parity (Price_ppp) and Price of a 20 cigarette pack of the most sold brand in US$ at official exchange rates (Price _US$). The data of HDI and the information of tobacco were mined from WHO and United Nations Development Programme websites, respectively [7], [8].

### 2.2 Analytical procedures

To study the correlation between HDI and the variables of the study, Pearson correlation coefficient was used. Meanwhile, linear regression analysis was used to analyze the relationship between HDI and the variables of the study.
